# Role for Egr1 in the Transcriptional Program Associated with Neuronal Differentiation of PC12 Cells

**DOI:** 10.1371/journal.pone.0170076

**Published:** 2017-01-11

**Authors:** Kenneth W. Adams, Sergey Kletsov, Ryan J. Lamm, Jessica S. Elman, Steven Mullenbrock, Geoffrey M. Cooper

**Affiliations:** 1 Department of Biological Sciences, Bridgewater State University, Bridgewater, Massachusetts, United States of America; 2 Department of Biology, Boston University, Boston, Massachusetts, United States of America; Hungarian Academy of Sciences, HUNGARY

## Abstract

PC12 cells are a well-established model to study how differences in signal transduction duration can elicit distinct cell behaviors. Epidermal growth factor (EGF) activates transient ERK signaling in PC12 cells that lasts 30–60 min, which in turn promotes proliferation; nerve growth factor (NGF) activates more sustained ERK signaling that lasts 4–6 h, which in turns induces neuronal differentiation. Data presented here extend a previous study by Mullenbrock et al. (2011) that demonstrated that sustained ERK signaling in response to NGF induces preferential expression of a 69-member gene set compared to transient ERK signaling in response to EGF and that the transcription factors AP-1 and CREB play a major role in the preferential expression of several genes within the set. Here, we examined whether the Egr family of transcription factors also contributes to the preferential expression of the gene set in response to NGF. Our data demonstrate that NGF causes transient induction of all Egr family member transcripts, but a corresponding induction of protein was detected for only Egr1 and 2. Chromatin immunoprecipitation experiments provided clearest evidence that, after induction, Egr1 binds 12 of the 69 genes that are preferentially expressed during sustained ERK signaling. In addition, Egr1 expression and binding upstream of its target genes were both sustained in response to NGF versus EGF within the same timeframe that its targets are preferentially expressed. These data thus provide evidence that Egr1 contributes to the transcriptional program activated by sustained ERK signaling in response to NGF, specifically by contributing to the preferential expression of its target genes identified here.

## Introduction

PC12 rat pheochromocytoma cells are an established model to study molecular events associated with neuronal differentiation. Following treatment with nerve growth factor (NGF), PC12 cells differentiate into neuron-like cells [1], characterized by cessation of cell proliferation, up-regulation of neuronal genes, neurite outgrowth, and development of electrical excitability. NGF initiates these effects through activation of its receptor TrkA, which in turn stimulates multiple signaling cascades including the Ras/Raf/MEK/ERK, PI 3-kinase/Akt, and phospholipase C pathways [2]. ERK signaling plays a central role in PC12 differentiation, indicated by experiments demonstrating that inhibition of ERK signaling pharmacologically or via expression of dominant-negative Ras, Raf, MEK, or ERK blocks differentiation in response to NGF [3–5], whereas expression of constitutively active forms of Ras, Raf, MEK, or ERK is sufficient to induce differentiation [6]. The mechanism through which ERK signaling induces PC12 differentiation, however, is not fully understood.

One key mechanistic link between ERK signaling and PC12 differentiation is the duration for which ERK remains active following stimulation. Stimulation with NGF induces rapid ERK activation that peaks within minutes and then gradually declines to basal levels through 3–4 h. Sustained ERK signaling through this timeframe is essential for differentiation, demonstrated in part by observation that some growth factors induce more transient ERK signaling and do not cause differentiation. The most well studied example is epidermal growth factor (EGF), which similarly stimulates ERK activation within minutes, but activity declines to basal levels more rapidly within 30–60 min. Rather than inducing differentiation, this more transient ERK signal promotes proliferation [7].

One potential basis for the distinct biological response of PC12 cells to NGF is that NGF elicits a transcriptional program that is dependent on sustained ERK signaling through 2–4 h after stimulation. To investigate this, DNA microarray analysis was used to compare global gene expression profiles of PC12 cells treated with either NGF or EGF for 2 or 4 hours, which identified a set of 69 genes preferentially up-regulated in response to NGF at those time points (of which many have known roles in neuronal differentiation and/or function) [8]. Subsequent experiments indicated that transcription factors AP-1 and CREB contribute to the preferential expression of several genes within the set. Here, we tested the hypothesis that Egr transcription factors likewise contribute to the preferential expression of the gene set, which was based on the presence of putative Egr binding sites upstream of 21 genes within the set.

The Egr family comprises five members—Egr1-4 and Wilms Tumor 1 (WT1)—which share highly homologous DNA-binding domains at their C-termini composed of three zinc finger motifs that bind similar GC-rich, 9-nucleotide response elements [9–15]. Their N-termini exhibit greater variability, but similarly contain activation domains through which they transactivate target genes [10, 12, 16–19]. Egr1-3 also contain an R1 domain that binds coregulators NAB1 and 2 [20–22]; NABs are best characterized as corepressors [23], but can also act as coactivators [24]. Egr4 and WT1 lack an R1 domain, however, WT1 also exhibits trans-activation and -repression activities in a target gene-specific manner [19, 25–29]. The molecular details that dictate the effect of Egr1-3 and WT1 on target gene expression—activation or repression—are not fully understood.

Egr proteins are also immediate early genes (IEGs) induced rapidly and transiently in response to several stimuli including growth factors, cytokines, neurotransmitters, and multiple cellular stressors (e.g., hypoxia and oxidative stress) [30–32]. Upon induction, Egr proteins contribute to several cell behaviors including proliferation, apoptosis, and differentiation in a cell type- and stimulus-specific manner [11, 33–35]. Roles for Egr1-3 are particularly well-documented in the nervous system, where Egr1 and 3 play important roles in learning and memory through regulation of genes that contribute to synaptic plasticity and long-term potentiation [36–50]. Egr2 is expressed in Schwann cells, where it promotes peripheral nerve myelination through, at least in part, activation of the gene encoding myelin protein zero [51–56].

A role for Egr proteins in PC12 differentiation is also established. Levkovitz et al. (2001) determined that NGF-induced neurite outgrowth is inhibited by transfection with a truncated Egr3 consisting of its DNA-binding domain that acts as a dominant-negative on Egr-mediated transactivation [57]. Transfection with Egr1 siRNA by Ravni et al. (2008) similarly inhibited neurite outgrowth induced by PACAP (pituitary adenylate cyclase-activating polypeptide) [58]. Identification of Egr transcriptional targets that contribute to PC12 differentiation has been limited to the gene encoding p35, which activates Cdk5 kinase activity via protein-protein interactions [59]. Harada et al. (2001) demonstrated that Egr1-mediated induction of p35 expression is necessary for Cdk5 activation and neurite outgrowth in response to NGF. The present study provides evidence that Egr1 contributes to PC12 differentiation via regulation of several additional transcriptional targets.

## Materials and Methods

### Cell culture

PC12 cells [3] were maintained in growth medium consisting of Dulbecco’s modification of Eagle’s medium (DMEM) supplemented with 10% fetal bovine serum (FBS) and 5% horse serum (HS) at 37°C, 10% CO_2_. For gene expression and Western blot experiments, 10^6^ cells were plated per 60-mm dish in growth medium and incubated for 24 h before medium was replaced with reduced serum medium (DMEM + 0.5% HS). For ChIP experiments, 6 x 10^6^ cells were plated per 150-mm dish in growth medium and incubated for 72 h before medium was replaced with reduced serum medium. After placing in reduced serum medium, cells were incubated another 24 h before treatment with 50 ng/ml NGF (EMD Millipore, 480275) or 25 ng/ml EGF (EMD Millipore, 324831).

### Real-time reverse transcriptase polymerase chain reaction (RT-PCR)

For gene expression experiments, total RNA was extracted using TRIzol Reagent (Thermo Fisher Scientific, 15596026) as follows. Cell cutlures were washed once with PBS and lysed in 500 μl of TRIzol, 100 μl of chloroform were added to each lysate, and samples then incubated for 3 min at room temperature. Samples were subjected to centrifugation at 12,000 x g for 15 min at 4°C and the aqueous phase collected. Two hundred μl of 2-propanol were added to each sample and samples incubated for 10 min at room temperature. Samples were subjected to centrifugation at 16,000 x g for 10 min at 4°C and the supernatants removed. RNA pellets were washed by adding 1 ml of 70% ethanol, vortexed briefly, and then subjected to centrifugation at 16,000 x g for 15 min at 4°C. The supernatants were removed and RNA pellets air dried for approximately 10 min. RNA pellets were then suspended in 50 μl of nuclease-free water and incubated at 60°C for 10 min before spectrophotometry to determine nucleic acid concentrations.

Reverse transcription reactions were conducted on 900 ng of nucleic acids from each sample using TaqMan Reverse Transcription kit (Life Technologies, N8080234), according to the manufacturer’s instructions. Real-time PCR reactions where then conducted using SYBR Select Mater Mix (Life Technologies, 4472908), according to the manufacturer’s instructions. Primer sequences used for real-time PCR are listed in S1 Table.

### Immunoblot

Following treatment with NGF or EGF, cells were washed twice with phosphate-buffered saline (PBS), lysed directly in 300 μl of 2X Laemmli buffer, and heated to 95–100°C for 5 min. Twenty μl of each lysate were loaded and electrophoresed through SDS-polyacrylamide gels (10% for Egr1 immunoblots, 12% for Egr2-4, WT1, and β-actin immunoblots), transferred to nitrocellulose membrane, and immunoblot conducted to detect Egr1 (Santa Cruz, sc-110, 1:1000 dilution), Egr2 (Covance, PRB-236P, 1:500 dilution), Egr3 (Cell Signaling, 2559, 1:1000 dilution; Santa Cruz, sc-191, 1:500 dilution; Sigma, SAB2104196, 1 μg/ml working concentration), Egr4 (Santa Cruz, sc-19868, 1:500 dilution), WT1 (Santa Cruz, sc-68880, 1:500 dilution) and β-actin (Sigma, A5441, 1:20,000 dilution).

Membranes were blocked in 5% nonfat dried milk in TBS + 0.2% Tween-20 (blocking buffer) for 30 min at room temperature and then incubated with primary antibody diluted in blocking buffer for 12–18 h at 4°C. Membranes were then washed three times for 15 min in TBS + 0.2% Tween-20 and incubated with secondary antibody (goat anti-rabbit HRP-conjugate, Bio-Rad, 172–1019, 1:3000 dilution; goat anti-mouse HRP-conjugate, Bio-Rad, 170–6516, 1:3000 dilution) diluted in blocking buffer for 1–2 h at room temperature. Membranes were washed three times for 15 min in TBS + 0.2% Tween-20 after which bands were detected by chemiluminesce (Western Lightning Plus-ECL, Enhanced Chemiluminescence Substrate, Perkin Elmer, NEL105001).

### Chromatin immunoprecipitation (ChIP)

ChIP assays were performed as previously described [60]. For each experimental condition, cells from three 15-cm plates were scrapped, pooled, and crosslinked in 1% formaldehyde for 10 min at 37°C. Cells were then subjected to centrifugation at 800 x g for 4 min at 25°C and resulting cell pellets were washed twice with 15 ml of cold PBS supplemented with protease inhibitors (1 mM PMSF, 1 μg/ml aprotinin, 1 μg/ml pepstatin A). Cells were lysed in 4 ml of PIPES lysis buffer (5 mM PIPES, 85 mM KCl, 0.5% NP-40, pH 8.1) supplemented with protease inhibitors, subjected to centrifugation at 800 x g for 5 min at 4°C, and resulting nuclear pellets washed twice with 15 ml cold PBS supplemented with protease inhibitors. Pellets were resuspended in 1.2 ml of PBS lysis buffer (1% NP-40, 0.5% sodium deoxycholate, 0.1% SDS in PBS) supplemented with protease inhibitors and subjected to sonication using a Branson Sonifier S-250D Digital Ultrasonic Cell Disrupter with four 30-sec pulses at 25% amplitude. Samples were subjected to centrifugation at 16,000 x g for 15 min at 4°C and supernatants collected. One hundred μl of the supernatant were transferred to a clean tube, combined with 200 μl high salt lysis buffer, and stored at -20°C for use as “input fractions” during subsequent real-time PCR. Remaining volumes from sonication were precleared by incubation with 80 μl of protein A agarose 50% slurry for 30 min at 4°C with mixing followed by centrifugation at 16,000 x g for 30–60 sec at 4°C. Supernatants were collected and separated into 350 μl volumes for immunoprecipitation (IP) using 5 μg of the following antibodies: Egr1 (Santa Cruz, sc-110), Egr2 (Covance, PRB-236P), Egr3 (Santa Cruz, sc-191), Egr4 (Santa Cruz, sc-19868), and WT1 (Santa Cruz, sc-68880). Samples were incubated with antibody for 12–18 h at 4°C after which 60 μl of protein A agarose 50% slurry were added and incubated for 2 h at 4°C. Samples were subjected to centrifugation at 16,000 x g for 30–60 sec at 4°C, supernatant discarded, and protein A agarose beads subjected to five sequential washes with 1 ml of the following buffers: [1] low salt wash buffer (20 mM Tris, 150 mM NaCl, 2 mM EDTA, 0.1% SDS, 1% Triton X-100, pH 8.1), [2] high salt wash buffer (20 mM Tris, 500 mM NaCl, 2 mM EDTA, 0.1% SDS, 1% Triton X-100, pH 8.1), [3] LiCl wash buffer (10 mM Tris, 1mM EDTA, 250 mM LiCl, 1% IGEPAL-Ca 630, 1% deoxycholic acid, pH 8.10, [4] and [5] Tris-EDTA, pH 8.0. Antigens were then eluted from protein A agarose by two sequentional incubations in 150 μl elution buffer (1% SDS, 100 mM sodium bicarbonate) for 15 min at room temperature, which were pooled into one 300 μl eluate for each sample/experimental condition. Each eluate and input fraction was incubated with 200 mM NaCl for 12–18 h at 65°C to reverse crosslinks, after which DNA was purfied using QIAquick Gel Extraction Kit (Qiagen, 28706); 900 μl Buffer QG and 300 μl 2-propanol were added to each sample for step 1, after which the procedure was conducted according to the manufacturer’s protocol. Real-time PCR was then conducted on 1:40 dilutions of input fraction DNA and 1:10 dilutions of IP fraction DNA using using SYBR Select Mater Mix (Life Technologies, 4472908) according to the manufacturer’s instructions and primers that anneal within 250 bp of respective predicted transcription factor binding sites (see S2 Table). Recovery of each binding site during IP was quantified as % input.

## Results

### Expression analysis of Egr family members in PC12 cells following NGF treatment

**Fig 1:**
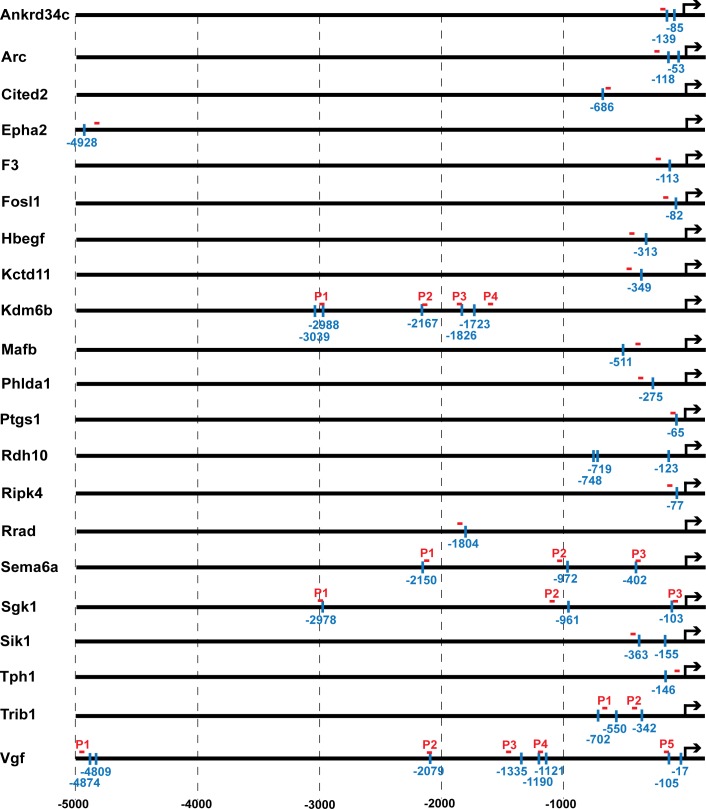
Predicted Egr binding sites and ChIP primer locations upstream of genes preferentially expressed during sustained ERK signaling in response to NGF. Of the 69 genes that Mullenbrock et al. (2011) determined are preferentially expressed during sustained ERK signaling in response to NGF, 21 contained putative Egr binding sites within 5 kb upstream of their transcription start site (TSS). The locations of each Egr binding site are denoted by the vertical blue lines. The red horizontal lines denote the relative locations of primer sets used for real time PCR to detect Egr binding to nearby Egr binding site(s). For genes with multiple dispersed Egr binding sites, multiple primers sets were designed (denoted P1, P2, etc.) to detect Egr binding to the nearest predicted Egr binding site.

Mullenbrock et al. (2011) identified 69 genes preferentially up-regulated in PC12 cells 2–4 h after NGF treatment as compared to EGF treatment; 21 of those genes contained putative Egr binding sites (identified using the V$KROX_Q6 position weight matrix) within 5 kb upstream of their transcription start sites that were conserved in human, mouse and rat (Fig 1 and S2 Table). Given the homology within their DNA-binding domains and the similarity of response elements to which they bind, all five Egr family members were examined as potential regulators of this gene set in response to NGF.

**Fig 2:**
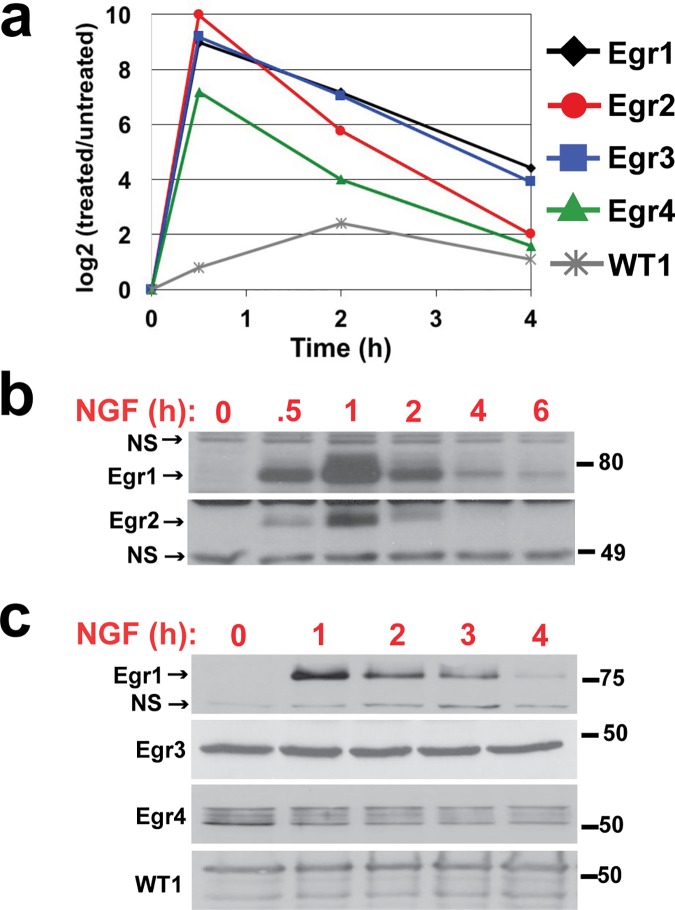
Time course analysis of Egr family expression following NGF treatment. PC12 cell cultures were treated with 50 ng/ml NGF for 0–4 h before harvest. **(a)** Total RNA was extracted and subjected to real-time RT-PCR to quantify changes in mRNA levels or **(b)** total cell lysates were prepared and subjected to SDS-PAGE and Western blot analysis to evaluate changes in protein levels. NS, nonspecific band.

Effects of NGF on Egr expression levels were evaluated first. Consistent with their characterization as IEGs, NGF induced a robust increase in Egr1-4 transcripts at 30 min post-treatment, followed by a gradual decline through 4 h (Fig 2A). Transcript levels of WT1 increased more modestly and slowly, peaking at 2 h, then declining through 4 h. Egr1 and 2 protein levels were affected similarly to their transcripts; Western blot for both proteins detected bands near their predicted molecular weights (82 kD predicted for Egr1, 50 kD predicted for Egr2) that rapidly accumulated through 1 h of NGF treatment and then gradually declined to near basal levels through 6 h (Fig 2B). Western blot analysis for Egr3, Egr4, and WT1 detected prominent bands near their predicted molecular weights (50 kD predicted for Egr3 and 4, 49 kD predicted for WT1) in untreated cells, all of which remained largely unchanged through 4 h of NGF treatment (Fig 2C). For Egr3 in particular, three different antibodies were used to evaluate its levels, all of which detected bands in the range of its predicted molecular weight and none of which exhibited a clear or consistent change in response to NGF (results from Sigma, SAB2104196 are shown in Fig 2C; data not shown from Cell Signaling, 2559 and Santa Cruz, sc-191). This expression analysis altogether demonstrates that NGF causes transient induction of mRNAs for all Egr family members and a corresponding induction of Egr1 and 2 protein; corresponding changes in Egr3, Egr4, and WT1 protein levels, on the other hand, were not detected.

Our data for Egr1 are consistent with previous studies that also demonstrated induction of Egr1 transcript and protein in PC12 cells with near identical kinetics following NGF treatment [59, 61]. Induction of Egr2-4 transcripts by NGF in PC12 cells has also been shown, however, these studies did not evaluate protein levels [10, 62, 63]. To our knowledge, effects of NGF on WT1 expression in PC12 cells has not been previously described. Several studies have documented corresponding transcript and protein inductions for Egr family members in cells other than PC12 in response to a variety of growth factors [59, 64–69]; however, previous examples of Egr transcript induction without concomitant protein induction (as shown here for Egr3, Egr4, and WT1) have not been previously documented.

### Egr1 binds upstream of several predicted target genes following NGF treatment

Binding of Egr family members to upstream regions of genes with putative Egr binding sites was examined next in PC12 cells by ChIP. Primers were designed within 250 bp of each predicted Egr binding site to quantify their recovery by real-time PCR, except for the three putative sites upstream of *Rdh10* for which primer design was limited due to high GC content (see Fig 1 and S2 Table for primer locations and sequences). Several genes (*Vgf*, *Kdm6b*/*Jmjd3*, *Trib1*, *Sema6a*, and *Sgk1*) contained multiple dispersed Egr binding site candidates, for which multiple primer sets were used (denoted P1, P2, etc. for each target gene) to evaluate Egr binding within the respective regions. Primers to amplify a region approximately 100 bp upstream of the *Myog* gene, which lacks an Egr binding site, were used as a negative control for Egr binding.

**Fig 3:**
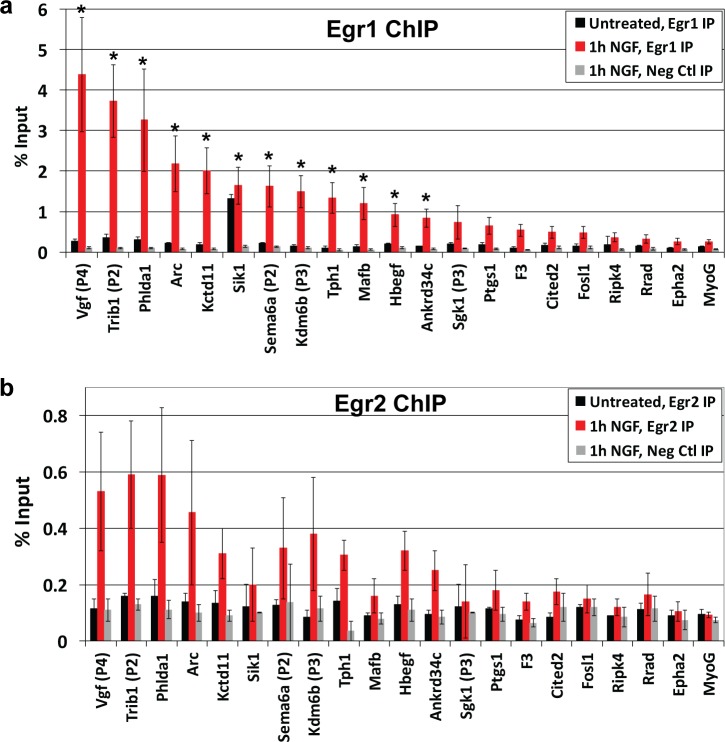
ChIP analysis to evaluate binding of Egr1 and Egr2 upstream of genes with predicted Egr binding sites. PC12 cultures were treated with or without 50 ng/ml NGF for 1 h and subjected to ChIP assay using antibodies against Egr1, Egr2, or an IgG control. Real-time PCR was then conducted on the immunoprecipitated DNA using primers within 250 bp of each predicted Egr binding site (see Fig 1 and S2 Table for primer locations and sequences). For genes with multiple dispersed Egr binding sites, multiple primer sets (denoted P1, P2, etc.) were used; only data for the set that detected highest level of binding are included here (see S1 Fig for data from the remaining primer sets). Primers to amplify a region approximately 100 bp upstream of the *Myog* gene were used as a negative control for Egr1 binding. **(a)** Data from Egr1 ChIP are plotted as % input and are averages from three to four independent experiments ± S.E. *, One-tailed Student’s *t* tests were conducted comparing the % input value for *Myog* after Egr1 IP to the % input values for each predicted Egr target after Egr1 IP, which yielded *p* values ≤ 0.05 for 12 predicted Egr targets. **(b)** Data from Egr2 ChIP are plotted as % input and are averages from two independent experiments ± S.E.

Binding of Egr1 was examined first due to its established role in PC12 differentiation [58, 59]. Since the data in Fig 2B show that Egr1 levels peak at 1 h post-NGF treatment, cells were treated with NGF for 0 or 1 h and subjected to ChIP with either anti-Egr1 antibody or non-immune IgG as a negative control; real-time PCR was then conducted on input and IP fractions to quantify recovery during IP as % input. As shown in Fig 3A, % input values for most predicted Egr targets were similar to *Myog* following Egr1 ChIP from untreated cells, whereas % input for *Sik1/Snf1lk* was 7-fold greater, indicating Egr1 is bound upstream of only *Sik1/Snf1lk* in the absence of NGF. After 1 h of NGF treatment, % input values increased well above that of *Myog* for several targets. Of the 20 genes examined, 15 exhibited average values greater than 2-fold above *Myog*, of which 12 were statistically significant (*p* < 0.05) and 2 approached significance (*p*-values for *Ptgs1* and *F3* from a one-tailed Student’s *t* test were 0.056 and 0.053, respectively). To our knowledge, 10 of these genes (*Trib1*, *Phlda1*, *Sema6*, *Kctd11*, *Sik1/Snf1lk*, *Kdm6b/Jmjd3*, *Mafb*, *Hbegf*, *Ankrd34c*, and *Ptgs1*) are novel Egr1 targets, whereas 5 (*Vgf*, *Arc*, *Tph1*, *Sgk1*, and *F3*) were previously identified targets [70–74]. For genes that were tested with multiple primer sets, data for the set with highest % input value are included in Fig 3; results from the remaining primer sets are provided in S1 Fig. These data indicate that Egr1 induction by NGF results in its binding upstream of approximately 17% of the genes that are preferentially expressed during sustained ERK signaling, whereas Egr1 is constitutively bound to *Sik1/Snf1lk*. The molecular explanation for Egr1’s differential binding to *Sik1/Snf1lk* versus the other targets was not examined.

ChIP with anti-Egr2 antibody yielded similar results to Egr1, but with notable differences (Fig 3B). In untreated cells, Egr2 binding was not detected for any targets, including *Sik1/Snf1lk*. After NGF treatment, 11 targets exhibited % input values greater than 2-fold above *Myog*, which were the same targets bound by Egr1 after NGF treatment excluding *Mafb*. However, values for those 11 targets were 3- to 8-fold lower than % input values from Egr1 ChIP. Whether these differences reflect differential binding of Egr1 versus Egr2 upstream of target genes or differential effectiveness of the respective antibodies in ChIP was not determined. ChIP with anti-Egr3, -Egr4 or -WT1 antibodies did not demonstrate binding of these family members upstream of any putative targets before or after NGF treatment (data not shown).

Altogether, these ChIP data demonstrate that NGF induces Egr1 binding upstream of several genes that are preferentially up-regulated during sustained ERK signaling, indicating it may contribute to their expression at 2–4 h. The data provide more modest evidence for Egr2 binding, suggesting it may also play a role. Given the more robust data for Egr1, additional experiments focused on further examining its role in the network.

### NGF induces sustained Egr1 expression and binding to target genes associated with PC12 neuronal differentiation

**Fig 4:**
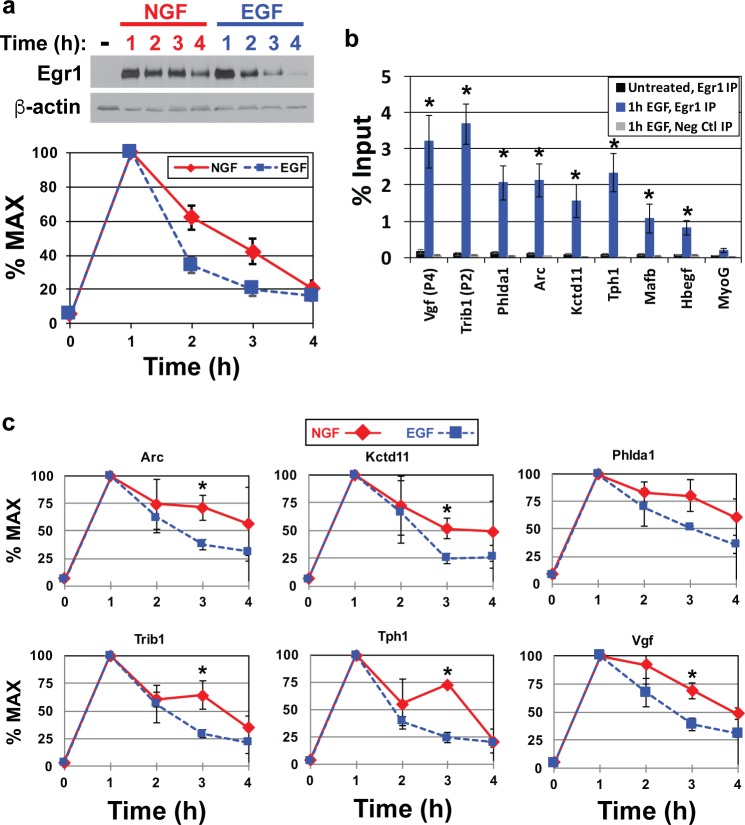
Sustained Egr1 expression and binding upstream of target genes in response to NGF versus EGF. **(a)** PC12 cell cultures were treated with 50 ng/ml NGF or 25 ng/ml EGF for 0–4 h. Total cell lysates were harvested and subjected to SDS-PAGE and Western blot for Egr1. Egr1 levels were quantified by densitometry and converted to % maximum levels for ten independent experiments and plotted (average ± S.E.). **(b)** PC12 cultures were treated with or without 25 ng/ml EGF for 1 h and subjected to ChIP assay using antibodies against Egr1 or an IgG control. Real-time PCR was then conducted on the immunoprecipitated DNA using primers within 250 bp of predicted Egr binding sites (see Fig 1 and S2 Table for primer locations and sequences). Primers to amplify a region approximately 100 bp upstream of the *Myog* gene were used as a negative control for Egr1 binding. Data are plotted as % input and are averages from three to six independent experiments ± S.E. *, One-tailed Student’s *t* tests were conducted comparing the % input value for *Myog* after Egr1 IP to the % input values for each predicted Egr target after Egr1 IP, which yielded *p* values ≤ 0.05. **(c)** PC12 cell cultures were treated with 50 ng/ml NGF or 25 ng/ml EGF for 0–4 h and subjected to ChIP with anti-Egr1 antibody. Immunoprecipitated DNA was then subjected to real-time PCR with primers to detect Egr1 binding to a subset of its target genes, which was quantified as % input. Percent input values were then converted into % maximum values, which were plotted. *, One-tailed Student’s *t* tests were conducted comparing the % input values at each time point after NGF versus EGF treatment, which yielded *p* values ≤ 0.05 at the 3-hour time point for *Arc*, *Kctd11*, *Trib1*, *Tph1*, and *Vgf*.

To begin examining how Egr1 might contribute to the preferential expression of its target genes 2–4 h post-NGF versus -EGF treatment, Western blot analysis and densitometry were conducted to compare its levels 0–4 h after treatment with each growth factor. Both NGF and EGF induced strong up-regulation of Egr1 at 1h followed by a decline to near basal levels through 2–4 h (Fig 4A); however, Egr1 declined more rapidly after 1 h of EGF treatment. This is consistent with a previous study by Harada et al. (2001), who also demonstrated sustained Egr1 expression through 3 h of NGF treatment compared to EGF; moreover, the authors showed that sustained Egr1 levels caused sustained expression of p35, which in turn promoted differentiation via sustained activation of Cdk5 [59]. These observations together with those from Fig 3A suggest that sustained Egr1 levels in response to NGF may result in sustained binding and regulation of its target genes.

**Fig 5:**
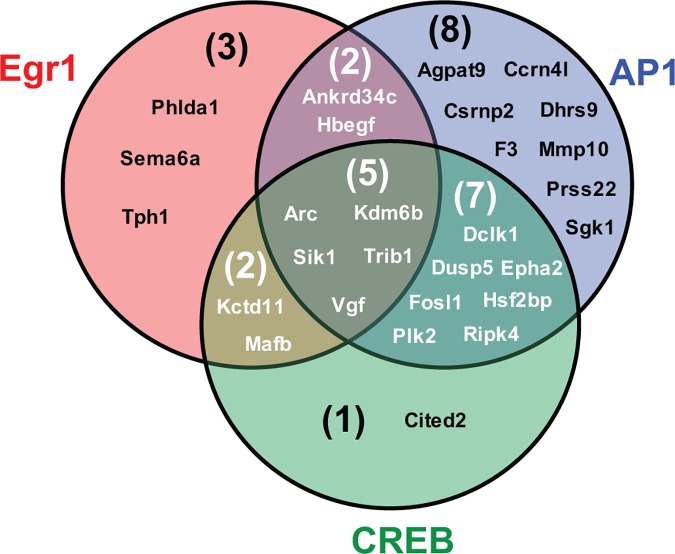
AP-1, CREB, and Egr1 cooperatively regulate 28 genes during their preferential expression in response to NGF and sustained ERK signaling. The Venn diagram summarizes genes bound by AP-1, CREB, and/or Egr1 during their preferentially expression in response to NGF and sustained ERK signaling, as detected by Mullenbrock et al. (2011) and the present study.

To test this, ChIP was conducted to first determine if Egr1 similarly binds a subset of predicted targets in response to EGF when at peak levels 1 h after treatment (Fig 4B). As expected, % input values for all tested targets were similar to *Myog* after Egr1 IP from untreated cells, whereas values increased above *Myog* after EGF treatment. Comparison of % input values for each gene independently after 1 h NGF versus 1 h EGF treatment showed no significant difference (S3 Table), indicating Egr1 binds its targets at similar levels after 1 h of treatment with either growth factor. Next, maintenance of Egr1 binding upstream several targets at 1–4 h post-treatment was evaluated by ChIP. For these experiments, the time point with highest % input value was defined as maximum binding and values for remaining time points were converted to % maximum. Similar to its protein levels, Egr1 binding to all targets peaked at 1 h post-treatment with either NGF or EGF and then gradually declined through 4 h (Fig 4C). However, binding was comparatively sustained after NGF treatment, specifically at the 2 and 3 h time points, which mirrors changes in Egr1 protein levels. These data confirm that NGF induces sustained expression and binding of Egr1 to several target genes that are preferentially expressed during sustained ERK signaling and more broadly supports the hypothesis that Egr1 acts cooperatively with CREB and AP-1 to activate a transcriptional program that is associated with sustained ERK signaling in response to NGF (Fig 5).

## Discussion

The experiments described here extend a previous study aimed at understanding how differences in ERK signaling duration cause distinct behavioral responses in PC12 cells; more specifically, how sustained ERK signaling lasting 3–4 h in response to NGF induces neuronal differentiation, whereas more transient ERK signaling lasting 30–60 min in response to EGF promotes proliferation [8]. The previous study identified a transcriptional program that is activated by sustained ERK signaling in response to NGF, in which CREB and AP-1 act as part of a transcription factor network that preferentially up-regulates a set of 69 genes 2–4 h after treatment with NGF versus EGF. The present study tested the hypothesis that Egr proteins (Egr1-4 and WT1) act cooperatively with CREB and AP-1 to activate this gene set, which is based on computational analysis that identified putative Egr binding sites upstream of 21 genes within the set of 69. Of the five Egr family members, our data provides strongest evidence that Egr1 contributes to this transcriptional program.

As expected based on its previous characterizations as an IEG, NGF induced strong, but transient Egr1 expression; protein levels peaked 1 h after treatment and then declined to near basal levels through 6 h. ChIP experiments demonstrated that 1 h of NGF treatment induced Egr1 binding upstream of 11 of the 21 genes with predicted Egr binding sites, whereas Egr1 was bound upstream of one target, *Sik1/Snf1lk*, at similar levels in untreated and NGF-treated cells. To our knowledge, nine of these 12 genes (*Trib1*, *Phlda1*, *Sema6*, *Kctd11*, *Sik1/Snf1lk*, *Kdm6b/Jmjd3*, *Mafb*, *Hbegf*, and *Ankrd34c*) are novel Egr1 targets and three (*Vgf*, *Arc*, and *Tph1*) were previously identified. The kinetics of Egr1 binding to a subset of these targets were examined 0–4 h after NGF treatment; consistent with its expression pattern, binding peaked 1 h after NGF treatment and then gradually declined through 4 h. EGF induced similar effects on Egr1 expression and binding upstream of target genes, but with notable differences. Both Egr1 protein levels and binding upstream of targets peaked 1 h after EGF treatment; however, both then declined more rapidly through 2–3 h compared to NGF treatment. These data collectively demonstrate that, compared to EGF, NGF induces sustained Egr1 expression and binding upstream of several target genes during the same timeframe in which those genes are preferentially expressed, which supports our hypothesis that Egr1 contributes to the transcriptional program activated by sustained ERK signaling. Given Egr1’s transactivation activity, the simplest extrapolation is that Egr1 contributes to the program through sustained transactivation of its target genes. However, since Egr1 can also act as a transcriptional repressor, a more complex mechanism for Egr1’s contribution cannot be ruled out.

The Venn diagram in Fig 5 summarizes our data and model for the cooperative roles of AP-1, CREB, and Egr1 in regulation of transcriptional targets during PC12 differentiation. Of the 69 genes preferentially up-regulated during sustained ERK signaling in response to NGF, 28 are bound by one or more of these three transcription factors within the same timeframe. AP-1 binds the largest subset (21 genes), of which 16 are also bound by Egr1 and/or CREB. Of the 12 Egr1 targets identified here, nine are similarly bound by AP-1 and/or CREB; and of the 15 CREB targets, all but one are also bound by AP-1 and/or Egr1.

One surprising result from the Egr1 ChIP experiments discussed here was the selective binding of Egr1 upstream of only *Sik1/Snf1lk* in the untreated cells, especially given its low expression levels compared to 1 h post-NGF treatment. AP-1, whose expression and binding upstream of target genes are induced by NGF similarly to Egr1 [8], also binds upstream of *Sik1/Snf1lk*. However, in contrast to Egr1, AP-1 binding to *Sik1/Snf1lk* was induced similarly to AP-1 binding to its other targets. Nevertheless, selective binding of transcription factors to specific target genes when at basal levels is not unprecedented. For example, transcription factors c-Myc, REST/NRSF, and Ste12, have been shown to selectively bind target genes when at basal levels [75–77]. Enrichment of CpG islands in the vicinity of binding sites has been proposed as a factor that promotes selective binding of transcription factors to sites via epigenetic modifications [76]; however, comparison of sequences surrounding the Egr1 binding sites identified in this study did not reveal a unique enrichment of CpG islands in the vicinity of the two putative Egr binding sites upstream of *Sik1/Snf1lk*.

Of the 69 genes preferentially induced by NGF, approximately 50% have been implicated in neuronal development and/or function [8], which is consistent with the Egr1 targets identified here. Of the 12 Egr1 targets, eight have previously characterized roles in the nervous system. *Vgf* and *Hbegf* both encode neurotrophic factors expressed throughout the nervous system and have been implicated in several aspects of its development and maintenance [71, 78–81]. *Kdm6b/Jmjd3* encodes an H3K27 demethylase and *Kctd11* encodes an adaptor for the ubiquitin ligase Cullin3, both which contribute to differentiation of embryonic and neural stem cells to neurons [82–86]. *Sema6a* encodes the transmembrane protein semaphorin6a, which regulates axon guidance and dendritic growth during development [87–89], whereas *Arc* encodes a post-synaptic protein that plays important roles in synaptic plasticity associated with learning and memory [90, 91]. *Mafb* encodes a transcription factor that controls hindbrain segmentation and development at least in part via regulation of *Hoxa3* and *Hoxb3* expression [92–94]. *Tph1* encodes tryptophan hydroxylase-1, which catalyzes the rate-limiting step in serotonin synthesis; it is expressed in mouse brain during late stages of development and then restricted to the pineal gland in adulthood. Polymorphisms within the *Tph1* gene have been implicated in multiple psychiatric conditions [95–97].

Examination of Egr family members other than Egr1 provided more modest evidence for contribution of Egr2 to the transcriptional program. Egr2 transcript and protein levels mirrored those of Egr1 in response to NGF. ChIP experiments suggested induction of Egr2 binding to predicted targets after NGF treatment with an overall pattern similar to Egr1. Target genes that yielded highest % input values from Egr1 ChIP after 1 h of NGF treatment (e.g., *Vgf*, *Trib1*, and *Phlda1*) also yielded highest values from Egr2 ChIP. However, the Egr2 ChIP values were overall much lower (3- to 8-fold) than those from Egr1 ChIP, which could be due either to lower levels of Egr2 binding to the Egr binding sites or lower efficiency of the Egr2 antibody for ChIP in general. Given the modesty of the Egr2 ChIP results, additional experiments focused on Egr1.

Altogether, the experiments described here extend the study by Mullenbrock et al. (2011) that characterized a transcriptional program in PC12 cells whose activity is associated with sustained ERK signaling and neuronal differentiation. Mullenbrock et al. identified a set of 69 genes preferentially up-regulated during sustained ERK signaling and provided evidence that transcription factors AP-1 and CREB cooperatively contribute to the preferential expression of 25 genes within the set. Data shown here provide evidence that transcription factor Egr1 also plays a cooperative role through regulation of 12 genes within the set, nine of which are also targeted by AP-1 and/or CREB during sustained ERK signaling (see Fig 5); we also identify nine novel targets of Egr1 regulation in general. Identification of additional transcription factors that contribute to the transcriptional program associated with PC12 neuronal differentiation awaits future experiments.

## Supporting Information

S1 Fig ChIP analysis to evaluate binding of Egr1 and Egr2 upstream of genes with multiple predicted Egr binding sites. PC12 cultures were treated with or without 50 ng/ml NGF for 1 h and subjected to ChIP assay using antibodies against Egr1, Egr2, or an IgG control. Real-time PCR was then conducted on the immunoprecipitated DNA using primers within 250 bp of each predicted Egr binding site (see Fig 1 and S2 Table for primer locations and sequences). For genes with multiple dispersed Egr binding sites, multiple primer sets (denoted P1, P2, etc.) were used (see S1 Fig for data from the remaining primer sets). Primers to amplify a region approximately 100 bp upstream of the *Myog* gene were used as a negative control for Egr1 binding. **(a)** Data from Egr1 ChIP are plotted as % input and are averages from three to four independent experiments ± S.E. *, Student’s *t* tests were conducted comparing the % input value for *Myog* after Egr1 IP to the % input values for each predicted Egr target after Egr1 IP, which yielded *p* values ≤ 0.05 for several primer sets. **(b)** Data from Egr2 ChIP are plotted as % input and are averages from two independent experiments ± S.E.
(TIFF)

S1 Table Primer sequences used for real-time RT-PCR. Transcript levels of Egr1-4 and WT1 were evaluated by real-time RT-PCR using the forward and reverse primers listed within this table.
(XLSX)

S2 Table Putative Egr binding site locations and ChIP primer sequences and locations. This table provides the locations and sequences of putative Egr binding sites identified within the rat genome. Locations and sequences for forward and reverse primers used in real-time PCR following ChIP are also listed, alongside their Figs 1 and 3 and S1 Fig designations.
(XLSX)

S3 Table Comparison of % input values from Egr1 ChIP following 1 h of NGF or EGF treatment. Percent input values for several Egr1 target genes after Egr1 ChIP from cells treated with 50 ng/ul NGF or 25 ng/ul EGF for 1 h are listed and compared by Student’s *t* test. *p* values for all Egr1 targets were ≥ 0.25.
(XLSX)
