# Supplementary figures and images for: Role for Egr1 in the Transcriptional Program Associated with Neuronal Differentiation of PC12 Cells
Source: PLoS One. 2017 Jan 11;12(1):e0170076. doi: 10.1371/journal.pone.0170076 (PMC5226839; doi:10.1371/journal.pone.0170076)

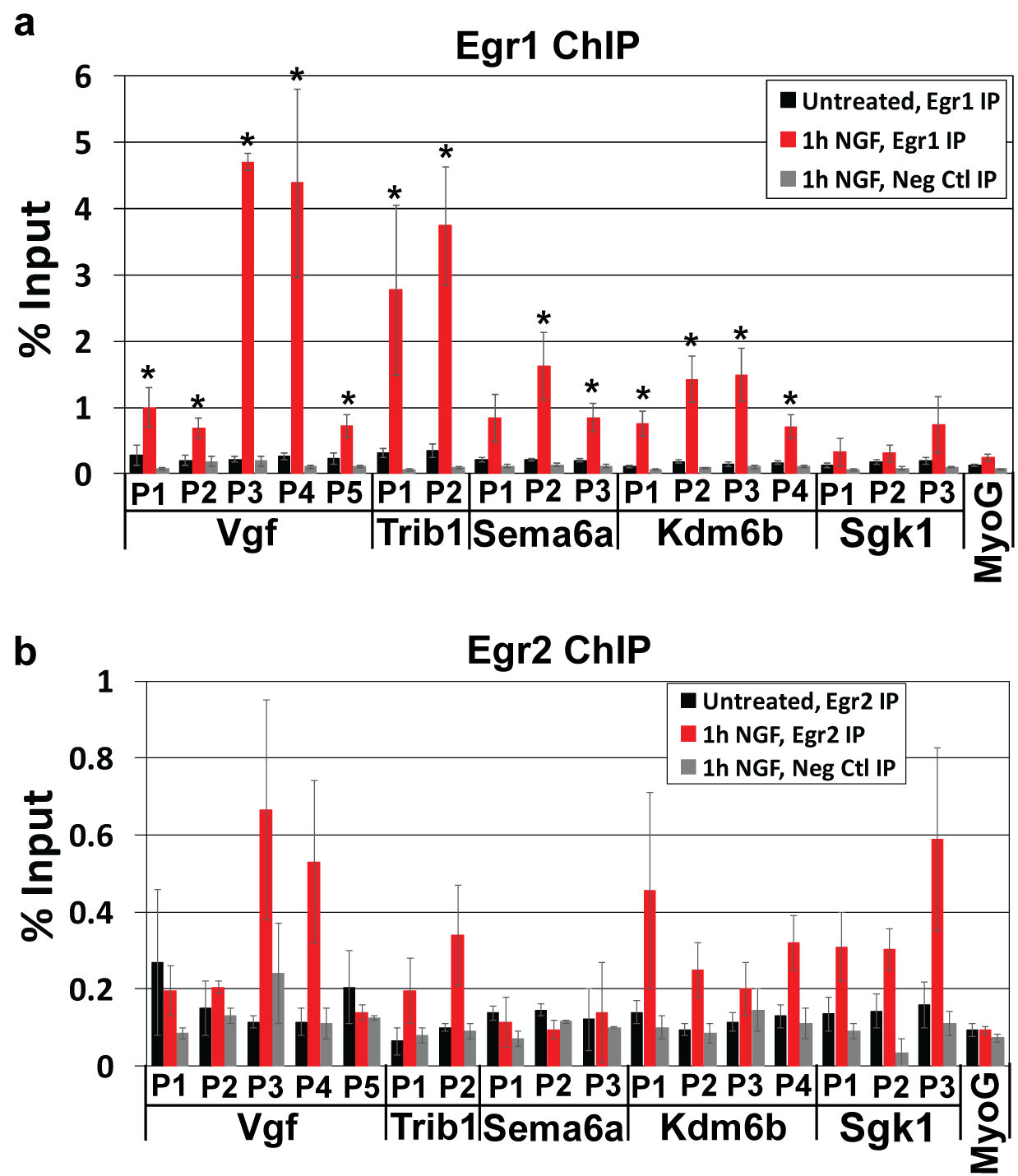

Supplement: S1 Fig — PC12 cultures were treated with or without 50 ng/ml NGF for 1 h and subjected to ChIP assay using antibodies against Egr1, Egr2, or an IgG control. Real-time PCR was then conducted on the immunoprecipitated DNA using primers within 250 bp of each predicted Egr binding site (see Fig 1 and S2 Table for primer locations and sequences). For genes with multiple dispersed Egr binding sites, multiple primer sets (denoted P1, P2, etc.) were used (see S1 Fig for data from the remaining primer sets). Primers to amplify a region approximately 100 bp upstream of the Myog gene were used as a negative control for Egr1 binding. (a) Data from Egr1 ChIP are plotted as % input and are averages from three to four independent experiments ± S.E. *, Student’s t tests were conducted comparing the % input value for Myog after Egr1 IP to the % input values for each predicted Egr target after Egr1 IP, which yielded p values ≤ 0.05 for several primer sets. (b) Data from Egr2 ChIP are plotted as % input and are averages from two independent experiments ± S.E. (TIFF) [file pone.0170076.s001.tiff]
